# The synergistic effects of short inter-pregnancy interval and micronutrients deficiency on third-trimester depression

**DOI:** 10.3389/fnut.2022.949481

**Published:** 2022-09-28

**Authors:** Jing Lin, Ye Zhou, Wei Gu

**Affiliations:** ^1^International Peace Maternity and Child Health Hospital, School of Medicine, Shanghai Jiao Tong University, Shanghai, China; ^2^Shanghai Key Laboratory of Embryo Original Diseases, Shanghai, China; ^3^Shanghai Municipal Key Clinical Specialty, Shanghai, China

**Keywords:** inter-pregnancy interval, folic acid, vitamin D, depression, nomogram

## Abstract

**Objective:**

To explore the effect of inter-pregnancy interval (IPI) and micronutrients on depression in the third trimester of pregnancy.

**Materials and methods:**

A total of 5,951 eligible pregnant women were included in this single-center retrospective cohort study. Variables with potential effects on third-trimester depression were collected. These variables included: maternal factors [age, pregnancy interval, body mass index (BMI), BMI change, gravidity, native place, education, smoking, and alcohol consumption], previous delivery outcomes [preterm birth, preeclampsia, intrahepatic cholestasis of pregnancy (ICP), gestational diabetes mellitus (GDM), fetal growth restriction (FGR), and delivery mode], and micronutrients in early pregnancy (folic acid, 25-hydroxy vitamin D, vitamin B12, calcium, and ferritin). Univariate and multivariate analyses were used to screen the factors affecting the occurrence of depression. Based on these factors, the nomogram model was established. At the same time, the interaction between IPI and micronutrients was verified.

**Results:**

The incidence of depression in the third trimester of pregnancy was 4.3%. Univariate and multivariate analysis showed that there were five independent risk factors for third-trimester depression: gravidity, previous cesarean section delivery, folic acid, and vitamin D levels in early pregnancy and IPI. According to the multivariate logistic regression analysis, the prediction model and nomogram were established. The prediction cut-offs of the corresponding factors were calculated according to the Youden index. Finally, the synergistic effect of short IPI and micronutrient deficiency was verified.

**Conclusion:**

There is a synergistic effect between short IPI and micronutrient deficiency in early pregnancy, which can aggravate the occurrence of depression in late pregnancy.

## Introduction

Depression is a common complication during pregnancy and postpartum (1). In developed countries, the prevalence of perinatal depression is estimated to be 6.5–12.9% (2, 3). Depression during pregnancy is related to many complications and has a great adverse effect on mothers and infants, including preterm birth (4), low birth weight, fetal growth restriction (FGR) (4, 5), hypertension, preeclampsia, and gestational diabetes mellitus (GDM) (6). Perinatal depression is considered to be the result of a complex interaction involving genetics, epigenetics, neuroendocrine, hypothalamic pituitary adrenal axis, and environmental and social factors. Studies have suggested that untreated depression can promote the imbalance of the hypothalamic-pituitary-adrenal (HPA) axis and the release of stress hormones such as cortisol and catecholamine, resulting in placental hypoperfusion, subsequent intrauterine growth retardation, and preterm birth (6). Many controlled studies have shown that maternal depression can affect fetal development by changing the serotonin system and hormone reactivity of the HPA axis, especially corticotropin-releasing hormone (7), and even change the anatomical structure of the large brain of infants in the early stage of life (6), resulting in offspring cognition Impaired behavioral and emotional development and slow social and communication skills. Therefore, untreated perinatal depression will have serious consequences for mothers and their children, families, and the whole society. It will be a serious public health problem, and the consequences will even affect generations.

Inter-pregnancy interval (IPI) refers to the period between the birth of the previous child and the conception of the index child. Evidence suggests that short IPI and some micronutrient deficiencies may cause the development of depression through psychoneuroimmunology mechanisms. Women of childbearing age are particularly vulnerable to malnutrition because pregnancy and lactation are the main sources of nutritional stress in the body. In fact, during pregnancy or lactation, the demand for many nutrients will reach the highest level in a lifetime. High demands combined with insufficient intake often lead to nutritional deficiencies during pregnancy and breastfeeding (8). Continuous pregnancies, especially those with short IPIs, may complicate the problem and lead to multiple marginal nutritional deficiencies (9). The effects of IPI and early pregnancy nutrients on maternal mental health have not been explored to a great extent. China’s one-child policy was replaced by the new comprehensive two-child policy in 2015 (10). All couples can have two children, providing an ideal environment for the impact of IPI and early pregnancy nutrients on perinatal depression. In consideration that nutritional interventions are low cost, safe, easy to manage, and generally accepted by patients, it is necessary to pay more attention to the nutritional factors in mental health. Interventions to improve nutrition and optimize IPI are particularly important in women of childbearing age. Our study aims to explore the effect of IPI and micronutrients on depression in the third trimester of pregnancy. Deepening the understanding of these mechanisms is of great significance for guiding future research, clinical practice, and health education of perinatal depression (11).

## Materials and methods

### Participants

This retrospective cohort study was conducted at the International Peace Maternity and Child Health Hospital (IPMCH), a tertiary first-class obstetrics and gynecology hospital in Shanghai, China. We collected the data of pregnant women who gave birth in IPMCH twice in a row and the second delivery time was from April 2014 to March 2020. After excluding cases that did not meet the inclusion criteria or had missing data, we finally included 5,951 cases. The ethics committee of the International Peace Maternal and child health hospital approved the study procedure (Reference Number: GKLW 2021–23). All participants provided written informed consent, and the ethics committee approved the consent procedure.

### Inclusion and exclusion criteria

Women who met the following criteria were included: (1) singleton pregnancy; (2) women with both first and second births at the IPMCH; (3) regular obstetrics with available first-trimester serology; (4) third trimester Edinburgh score available; and (5) delivery gestational age ≥ 28 weeks.

Exclusion criteria were: (1) multiple pregnancy; (2) women with a history of depression or taking antidepressants before pregnancy; (3) parity ≥ 3; (4) stillbirth or miscarriage; (5) delivery gestational age < 28 weeks. (6) lack of serological markers or Edinburgh score; and (7) incomplete data. Ultimately 5,951 pregnant women participated in the study.

### Depression screening tool and Edinburgh postnatal depression scale diagnostic threshold determination

Several studies have shown that screening can improve pregnancy outcomes in depressed mothers (12, 13). Over the past few years, some organizations have updated their guidelines for screening for depression during pregnancy and postpartum. The United States Preventive Services Task Force (USPTF) and the American College of Obstetrics and Gynecology (ACOG) (1) recommend the use of validated standardized tools to screen pregnant women for depression during the perinatal period and provide sufficient resources for treatment and follow-up. Various validated patient questionnaires are effective screening tools for pregnant and postpartum women (2). Edinburgh Postnatal Depression Scale (EPDS) was launched in 1987 as a screening tool in the outpatient environment and has been widely used. It includes 10 questions about symptoms in the past week, with a score of 0–3 for each question and a total score of 0–30, which can be completed in 5 min to screen for possible depression. The self-reported EPDS (14) was developed to identify postpartum depression (PPD), but can also be used to screen for depression during pregnancy. EPDS is one of the simplest and most reliable screening tools, which has been proven to have good sensitivity and specificity. The study found that EPDS was twice as effective as clinician interviews in detecting depression (15). Levis et al. found that EPDS 11 as a cut-off value can maximize the comprehensive sensitivity and specificity, improve health outcomes and minimize hazards and resource use (16). This standard was established to avoid false negatives and identify the majority of patients who met the diagnostic criteria. In addition, there was no significant difference in accuracy according to the reference standard or participant characteristics, including whether EPDS was administered during pregnancy or postpartum. Therefore, EPDS was used to screen for third-trimester depression in this study, and 11 was used as the threshold of screening.

### Definition

IPI refers to the period between the birth of the previous child and the conception of the subsequent pregnancy. Gravidity is defined as the number of times that a woman has been pregnant. Parity is defined as the number of times that she has given birth to a fetus with a gestational age of 28 weeks or more, regardless of whether the child was born alive or was stillborn. Premature birth is defined as labor that happens before the 37th week of pregnancy. BMI (body mass index) is a person’s weight in kilograms divided by the square of height in meters. The EPDS is a set of 10-item self-report questions to screen women for symptoms of emotional distress during pregnancy and the postnatal period.

### Data collection procedures

All data during the two deliveries of participants were recorded by designated personnel and archived electronically, including age, gravidity, BMI, education level, place of residence, personal and family history, pregnancy interval, pregnancy complications, mode of delivery, and pregnancy outcome. Each participant had a regular prenatal examination in our hospital. The examination items in the early pregnancy included serum nutrients (i.e., folic acid, 25 hydroxyvitamin D, vitamin B12, calcium, and ferritin). During the late pregnancy, they completed the EPDS questionnaire to obtain the Edinburgh score.

### Blood samples collection

Fasting blood samples were collected from each woman after one night of random fasting (11–12 weeks of pregnancy) to detect micronutrients. Peripheral blood samples were collected in a BD vacuum container between 8 a.m. and 10 a.m. for treatment within 90 min. Plasma and serum aliquots were stored at –80°C for subsequent analysis.

### Study outcomes

The primary outcome measure is the occurrence of depression in the third trimester of pregnancy. All participants filled in the Edinburgh questionnaire within 2 weeks before delivery in the third trimester of pregnancy to obtain the EPDS score. The diagnostic threshold for depression in pregnancy is 11 points (16), 11 points and above are considered to be depressed pregnant women, and 11 points below are non-depression pregnant women.

The secondary outcome measures included peripheral blood micronutrient levels during early pregnancy (including folic acid, 25-hydroxyvitamin D, vitamin B12, calcium, and ferritin) and IPI. Maternal factors, previous delivery outcomes, and delivery mode were also collected. Maternal factors include age, IPI, BMI, BMI change, gravidity, native place, education, smoking, and alcohol consumption. Previous delivery outcomes included preterm birth, preeclampsia, ICP, GDM, and FGR. The pregnancy outcomes were recorded after delivery. IPI is defined as the period between the birth of the previous child and the conception of the index child. BMI changes represent the difference in weight between two consecutive early pregnancies.

### Statistical analysis

SPSS version 21.0 (IBM, Armonk, New York) and R software version 3.6.1 (R development core team, July 2019).^1^ Quantitative data were tested for normality. Those in line with the normal distribution were expressed as mean ± standard deviation and compared by independent sample *t*-test. And those not in line with the normal distribution are represented by the median and interquartile space. Qualitative data were expressed as ratios and compared by the chi-square test. The receiver operating characteristic (ROC) curve and the Youden index was used to determine the predictive cutoffs for each risk factor. Univariate logistic regression was used to find the related risk factors of depression during pregnancy. Multivariate regression analysis was used to adjust for confounding variables known to independently affect depression. In addition, we used a linear model to understand the interaction effects among the risk factors. Risk ratios (ORs) with 95% confidence intervals (CI) were calculated to identify risk factors and evaluate their impact. The nomogram was established based on the results of the multivariate logistic regression analysis using software R 3.0.3 by using the “rms” package. *P*-value < 0.05 is considered significant.

## Results

### Patient characteristics

**FIGURE 1:**
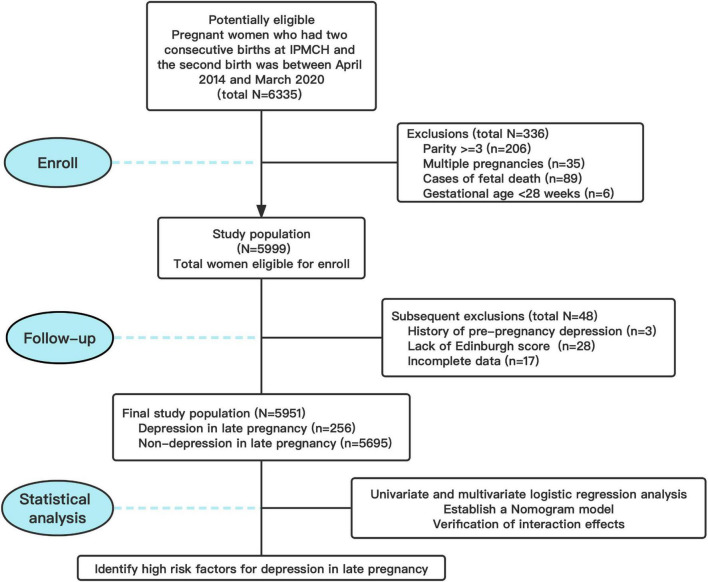
Flow chart illustrating study population selection and data availability.

Figure 1 shows the flow chart of inclusion and exclusion studies. Finally, 5,951 eligible pregnant women were included in the study. The incidence of third-trimester depression was seen in 256 cases. Pregnant women were divided into two groups according to whether they had depression or not. The baseline characteristics of patients in the two groups are shown in Table 1. Compared with the two groups, the incidence of depression was higher in pregnant women with gravidity ≥ 4 and those with previous cesarean section delivery (*P* < 0.001). In terms of micronutrients in early pregnancy, the levels of vitamin D, folic acid, and calcium in the depression group were significantly lower than those in the non-depression group (*P* < 0.05). In addition, the IPI in the depression group was significantly shorter than that in the non-depression group (*P* < 0.001). There was no significant difference between the two groups in age, native place, education level, smoking, alcohol consumption, complications of previous pregnancy, BMI, BMI change, vitamin B12, and serum ferritin in early pregnancy.

**TABLE 1:** Baseline characteristics of patients in the study.

			Non-depression (EPDS < 11, *n* = 5,695)	Depression (EPDS ≥ 11, *n* = 256)	*P*
**Maternal factors**					
	Age (*y*)		29.31 ± 3.15	29.22 ± 3.42	0.654
	Inter-pregnancy interval (months)		28.9 ± 14.6	23.4 ± 14.4	<0.001
	BMI (kg/m^2^)		20.9 ± 3.8	20.9 ± 3.4	0.969
	BMI change (kg/m^2^)		2.8 ± 0.3	3.4 ± 0.9	0.076
	Gravidity (%)	<4	5,086 (96.1%)	203 (3.9%)	<0.001
		≥4	609 (92.0%)	53 (8.0%)	
	Native place (%)	Urban	4,564 (95.7%)	208 (4.3%)	0.663
		Rural	1,131 (95.9%)	48 (4.1%)	
	Education (%)	High school and below	354 (96.6%)	12 (3.4%)	0.586
		Bachelor’s degree	3,887 (95.5%)	182 (4.5%)	
		Master’s degree or above	1,454 (95.8%)	62 (4.2%)	
	Smoking (%)	Non-smoking	5,674 (95.7%)	256 (4.3%)	0.331
		Smoking	21 (100%)	0 (0%)	
	Alcohol consumption (%)	Non-drinkers	5,634 (95.7%)	255 (4.3%)	0.294
		Drinkers	61 (98.4%)	1 (1.6%)	
**Outcomes of previous pregnancy**					
	Preterm birth (%)	No	5,508 (95.7%)	248 (4.3%)	0.889
		Yes	187 (95.9%)	8 (4.1%)	
	Preeclampsia (%)	No	5,562 (95.8%)	246 (4.2%)	0.138
		Yes	133 (93.0%)	10 (7.0%)	
	ICP (%)	No	5,651 (95.7%)	255 (4.3%)	0.417
		Yes	44 (97.8%)	1 (2.2%)	
	GDM (%)	No	5,180 (95.7%)	234 (4.3%)	0.911
		Yes	515 (95.9%)	22 (4.1%)	
	FGR (%)	No	5,660 (95.7%)	253 (4.3%)	0.223
		Yes	35 (92.1%)	3 (7.9%)	
	Delivery mode (%)	Vaginal delivery	3,878 (96.8%)	129 (3.2%)	<0.001
		Cesarean delivery	1,817 (93.5%)	127 (6.5%)	
**Trace element in early pregnancy**					
	Vitamin D (nmol/L)		46.8 ± 17.8	38.5 ± 16.2	<0.001
	Vitamin B12 (pmol/L)		376.2 ± 40.8	381.5 ± 51.3	0.552
	Folic acid (nmol/L)		27.9 ± 8.46	22.5 ± 10.2	<0.001
	Calcium (mmol/L)		2.3 ± 0.1	2.2 ± 0.1	0.003
	Serum ferritin (μg/L)		71.2 ± 9.9	72.6 ± 8.0	0.650

Data are given as number (percentage) or mean ± SD.

BMl, body mass index; GDM, gestational diabetes mellitus; ICP, intrahepatic cholestasis of pregnancy; FGR, fetal growth restriction.

### Incidence of depression in the third trimester of pregnancy and screening of risk factors

**FIGURE 2:**
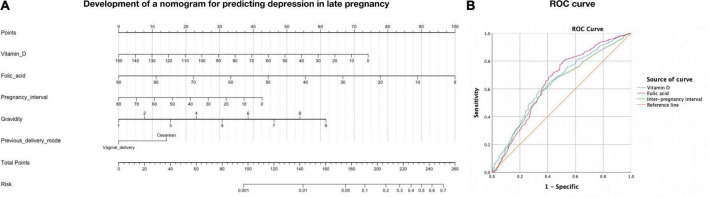
**(A)** A nomogram model to predict the incidence rate of third-trimester depression. To calculate the probability of depression in the third trimester, draw a vertical line on the corresponding axis of each risk factor until it reaches the top line marked “point,” summarize the points of all risk factors, and then draw a line down the axis marked “total points” until it intersects the lower line indicating the probability of depression. **(B)** ROC curves of relationships between the risk factors and the occurrence of depression.

The incidence of third-trimester depression was 4.3% in this study. The risk factors in the two groups were compared, and the results were shown in Table 1. The results showed that gravidity, previous delivery mode, the levels of vitamin D, folic acid, and calcium in the first trimester of pregnancy, and the IPI were related to the occurrence of depression. The results of univariate analysis and multivariate analysis were shown in Table 2. After adjusting the confounding factors, the following five factors were independent risk factors for the occurrence of depression: gravidity (OR = 2.633, 95% CI 1.846–3.756), previous delivery mode (OR = 7.095, 95% CI 3.490–14.421), vitamin D in early pregnancy (OR = 0.882, 95% CI 0.849–0.916), folic acid in early pregnancy (OR = 0.821, 95% CI 0.783–0.861), and IPI (OR = 0.836, 95% CI 0.792–0.883). The prediction model was developed based on these factors and presented as a nomogram (Figure 2A). The nomogram model was established to predict the incidence rate of depression during pregnancy based on the risk factors in the multivariable analysis. In addition, we compared the pregnancy outcomes of the two groups, including the incidence of premature delivery, FGR, macrosomia, neonatal asphyxia, postpartum hemorrhage, and cesarean section. The results were shown in Table 3. It could be found that the incidence of FGR and cesarean section was higher in depressed pregnant women (*P* < 0.05), while there was no difference in other pregnancy outcomes.

**TABLE 2:** The univariate and multivariate analysis for risk factors associated with depression during pregnancy.

Variables	Univariate analysis			Multivariate analysis*		
	OR	95% CI	*P*	OR	95% CI	*P*
**Gravidity**	1.710	1.351–2.166	<0.001	2.633	1.846–3.756	<0.001
**Delivery mode**	5.172	2.876–9.301	<0.001	7.095	3.490–14.421	<0.001
**Vitamin D**	0.892	0.866–0.919	<0.001	0.882	0.849–0.916	<0.001
**Folic acid**	0.823	0.792–0.856	<0.001	0.821	0.783–0.861	<0.001
**Calcium**	0.125	0.027–0.573	0.007	0.083	0.002–2.843	0.168
**Inter-pregnancy interval**	0.859	0.823–0.897	<0.001	0.836	0.792–0.883	<0.001

*Variables with *P* < 0.05 in the univariate analysis were included in the multivariate analysis.

OR, odds ratio; Cl, confidence interval.

**TABLE 3:** The comparison of pregnancy outcomes between the two groups.

Pregnancy outcomes	Non-depression (EPDS < 11, *n* = 5,695)	Depression (EPDS ≥ 11, *n* = 256)	*P*
**FGR (%)**	122 (2.1%)	13 (5.1%)	0.008*
**Premature delivery (%)**	274 (4.8%)	12 (4.7%)	0.928
**Macrosomia (%)**	352 (6.2%)	15 (5.9%)	0.834
**Postpartum hemorrhage (%)**	76 (1.3%)	4 (1.6%)	0.777
**Neonatal asphyxia (%)**	40 (0.7%)	0 (0%)	0.417
**Cesarean section (%)**	2,133 (37.5%)	114 (44.5%)	0.022*

*P*-values were given by the Chi-square tests.

*P < 0.05 was considered statistically significant.

### Assessment of the specific cutoff values for micronutrients and inter-pregnancy interval with depression

We used a receiver-operating characteristic (ROC) curve to determine the relationships between the risk factors and the occurrence of depression (Figure 2B). The level of vitamin D, folic acid, and IPI was closely related to depression (*p* < 0.001). The Youden index was used to calculate the pregnancy specificity and regional cutoff values of vitamin D, folic acid and pregnancy interval associated with the incidence of depression. According to the maximum value of the Youden index, the cut-off value of vitamin D in early pregnancy was 40.75 nmol/L, the cut-off value of folic acid was 25.55 nmol/L, and the IPI cut-off was 23.5 months.

### Logistic regression analysis and verification of prediction threshold

**FIGURE 3:**
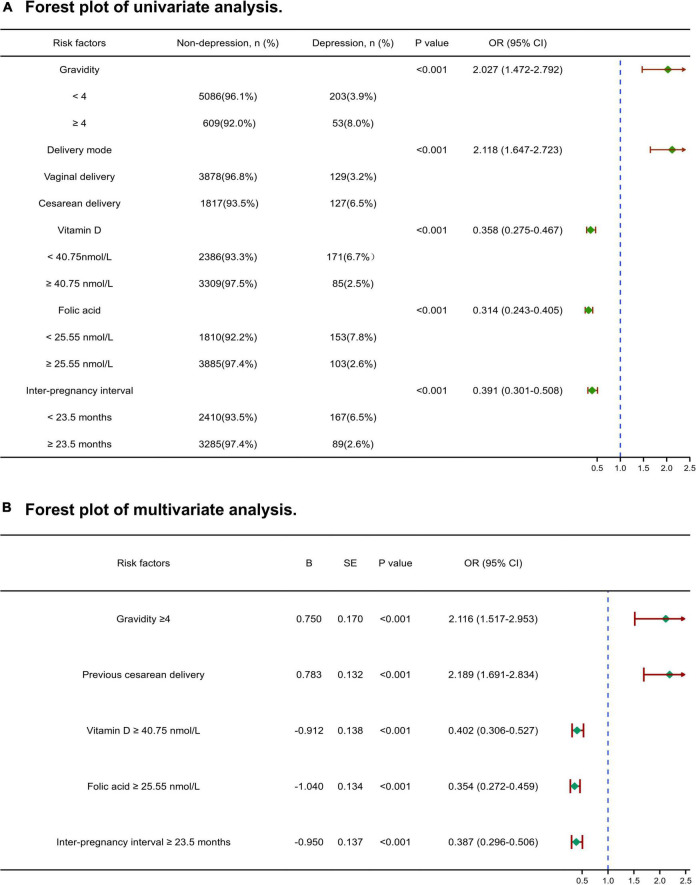
Forest plot of univariate and multivariate analysis for risk factors associated with third-trimester depression. **(A)** Univariate analysis, **(B)** multivariate analysis. OR, odds ratio; CI, confidence interval. A chi-square test and logistic regression analysis were used to identify the risk factors associated with depression during pregnancy.

Based on the above cut-off values, the levels of vitamin D and folic acid in early pregnancy, pregnancy interval, pregnancy times, and the mode of previous delivery were divided into two groups for univariate and multivariate logistic regression analysis. The analysis results and forest plots were shown in Figure 3. The results proved that the threshold calculated above has statistical value for predicting the occurrence of depression in the third trimester (*P* < 0.001). gravidity ≥ 4 (OR = 2.116, 95% CI 1.517–2.953) and previous cesarean delivery (OR = 2.189, 95% CI 1.691–2.834) are risk factors, while vitamin D ≥ 40.75 nmol/L (OR = 0.402, 95% CI 0.306–0.527), folic acid ≥ 25.55 nmol/L (OR = 0.354, 95% CI 0.272–0.459), and IPI ≥ 23.5 months (OR = 0.387, 95% CI 0.296–0.506) are protective factors.

### Analysis and visualization of interaction effects between inter-pregnancy intervals and micronutrients

**FIGURE 4:**
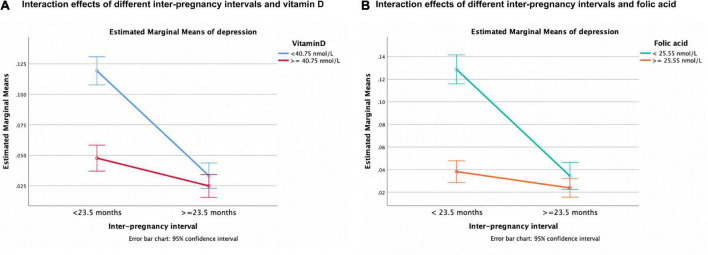
Interaction effects of different IPIs and vitamin D/folic acid levels on the risk of third-trimester depression. **(A)** Interaction effects of different inter-pregnancy intervals and vitamin D. **(B)** Interaction effects of different inter-pregnancy intervals and folic acid.

To explore the interaction effects between IPI and micronutrients in influencing depression, we established a linear model. The results were shown in Table 4. We found that there were significant interaction effects between them (*P* < 0.05). When IPI < 23.5 months, the levels of vitamin D, and folic acid in early pregnancy significantly affected the occurrence of depression (*P* < 0.001). On the contrary, when IPI ≥ 23.5 months, vitamin D and folic acid levels had little effect on depression (*P* > 0.05). Therefore, short IPI and deficiency of micronutrients in early pregnancy had a synergistic effect on depression, and the interaction effect was shown in Figure 4.

**TABLE 4:** Interaction effects between IPIs and trace elements on the risk of depression during pregnancy.

			Incidence		P*^a^*	OR	95% CI	P*^b^* for interaction
			Non-depression	Depression				
Inter-pregnancy interval	<23.5 months	Vitamin D <40.75 nmol/L	42.0%	74.7%	<0.001*	0.245	0.173–0.349	0.035*
		Vitamin D ≥40.75 nmol/L	58.0%	25.3%				
	≥23.5 months	Vitamin D <40.75 nmol/L	41.5%	50.5%	0.106	0.695	0.458–1.054	
		Vitamin D ≥40.75 nmol/L	58.5%	49.5%				
	<23.5 months	Folic acid <25.55 nmol/L	32.8%	69.5%	<0.001*	0.214	0.153–0.299	0.010*
		Folic acid ≥25.5 nmol/L	67.2%	30.5%				
	≥23.5 months	Folic acid <25.55 nmol/L	31.0%	40.7%	0.052	0.655	0.428–1.001		
		Folic acid ≥25.55 nmol/L	69.0%	59.3%								

OR, odds ratio; 95% CI, 95% confidence interval.

P*^a^* values were given by the Chi-square tests.

P*^b^* values for Interaction showed the interaction effect of inter-pregnancy interval and vitamin D/ folic acid on the risk of depression during pregnancy.

*P < 0.05 was considered statistically significant.

## Discussion

### Incidence of third-trimester depression

The incidence of depression in the third trimester of pregnancy in Shanghai was found to be 4.3% in this study. In previous studies, parity was found to be correlated with perinatal depression and nutrition (17, 18). Young and primipara are considered risk factors for depression during pregnancy (19). Therefore, in this study, in order to control the interference of parity and clarify the internal relationship between IPI and depression, the parity of all subjects was two. Depression rates vary from region to region due to regional economic, cultural, and policy diversity. Tang et al. (20) reported that the prevalence of depression in early pregnancy was 5.2% in Chongqing, China, and Tao et al. (21) reported that the prevalence of depression in the first and second trimesters of Anhui, China, was 4.7 and 3.6%, respectively. The above data are lower than those reported abroad (22), which showed the prevalence rates of depression in early, middle, and late pregnancy are 7.4, 12.8, and 12.0%, respectively. It is well known that due to differences in culture, customs and norms, Asians usually take a more conservative attitude than Westerners on some sensitive issues (23). The cultural differences may lead to the neglect of specific symptoms of Asian populations (24). These factors may lead to a low reporting rate of mental disorders in Chinese women. As one of the fastest growing cities, Shanghai women’s quality of life and education level is generally high. The distribution of perinatal depression in the Chinese mainland (25) indicates that the incidence rate of perinatal depression in Shanghai is lower than the average level. These factors make the incidence of depression in this study lower than that in many works of literature.

### Risk factors for third-trimester depression

Previous studies have shown that depression is associated with adverse reproductive events, such as cesarean section (19). Women experience high levels of post-traumatic stress, anxiety and depression after each miscarriage (26). Therefore, the frequency of abortion is closely related to the incidence rate of depression. But, quite apart from that, there is increasing evidence to support the provision of pre-pregnancy health care, and interventions to delay the age of first pregnancy, optimize birth spacing and provide perinatal micronutrient supplements that are expected to prevent the occurrence of depression during pregnancy. The results of this study also confirmed the above view. The five independent risk factors of third-trimester depression were gravidity, previous cesarean section delivery, folic acid, and vitamin D levels in early pregnancy and IPI.

### Effects of folic acid and vitamin D on depression

Adequate nutrients are necessary for many aspects of normal brain function (9) and the link between nutrition and mood has been fully proved. It is now considered that the consumption of nutritional reserves throughout pregnancy and postpartum nutritional delay may increase the risk of perinatal depression (27). Reduced postpartum micronutrient concentrations, especially folate levels, may take up to a year to return to normal folate values after delivery (28). Chong et al. (29) found that the plasma folate concentration of patients with prenatal depression was significantly lower than that of patients without depression. And poor folate status was associated with the severity and long-term onset of depression (30). Folic acid is essential for the synthesis and regeneration of tetrahydrobiopterin, which is an essential cofactor in the transformation of amino acid neurotransmitters (31). In addition, the lack of folate can lead to an increase of homocysteine concentration, affect DNA synthesis, repair, and methylation, and change the expression of regulatory genes of neural development (32). Studies have shown that point mutations in genes encoding key enzymes of folate metabolism are risk factors for depression (33). Folate deficiency is also related to drug resistance to antidepressant treatment (34), which will increase the recurrence rate of depression. In addition, Folic acid has been successfully used to strengthen the treatment of antidepressants in previous studies (35).

The role of vitamin D in calcium regulation and bone health has long been recognized, but its role in cell proliferation and development has been a subject of increasing attention (36), especially the link between vitamin D and psychoneuroimmunology, which is a hormone required for normal brain homeostasis and development (37). Many high-quality studies have shown (38, 39) that lower vitamin D levels may be associated with perinatal depression. The levels of serum 25 hydroxyvitamin D are a reliable measure of vitamin D, which is associated with several mood disorders. This mechanism may be related to the location of vitamin D receptors in the brain. After binding with VDR (vitamin D receptor), it can regulate the functions of several tissue cells in the body. In the brain, it promotes neurotransmission (40), neurogenesis, synaptogenesis, amyloid clearance, and prevents neuronal death (41). Vitamin D also has a protective effect on low levels of dopamine and serotonin (42). In the case of vitamin D deficiency, VDR filling is insufficient, which may interfere with the normal function of hormonal processes to prevent brain diseases, leading to emotional disorders and depression (43). Vitamin D supplementation has some ideal effects on the treatment of depression, especially when the serum vitamin D level is lower than the normal range (44). In the results of this study, calcium was originally statistically significant in univariate analysis, but its association with depression disappeared in multivariate analysis. It may be because vitamin D, as a confounding factor, is related not only to calcium but also to depression, thus causing a “false association” between calcium and depression. It is vitamin D level that is directly related to the onset of depression.

### Effect of short inter-pregnancy interval on depression

Some studies have shown that extending IPls may have beneficial effects, including reducing the risk of depression. Shorter IPIs may affect postpartum mood through two different mechanisms. The first is the social role and physical discomfort caused by frequent childbirth (45). Second, short birth spacing may damage maternal nutritional recovery and supplementary reserves. Many studies (46, 47) have shown that depression was associated with short IPI. Women’s malnutrition caused by pregnancy needs a certain time to supplement their nutritional reserves before the next pregnancy to obtain the best effect. Pregnancies with close intervals do not allow the mother enough recovery time to supplement essential nutrients, resulting in adverse pregnancy outcomes. This mechanism may be due to the failure to replenish maternal nutritional storage and re-establish immune balance. The results of this study also found that short IPI, low levels of folate and vitamin D were all independent risks for depression risk factors, and there is an interaction between them. In the case of short IPI, low levels of folic acid and vitamin D are more likely to lead to the occurrence of depression in pregnancy.

### Future prospect

Although screening is important for the detection of perinatal depression, the screening itself is not enough to improve results unless there are appropriate strategies to respond to positive screening results and further treatment and follow-up. If pregnancy spacing and micronutrient deficiency are identified as causal mechanisms for our findings, this will support the extension of pregnancy spacing and the promotion of dietary supplements in the postpartum and perinatal period (48), especially in low- and middle-income countries. The potential to improve mental health by changing diet is convincing. Nutritional interventions are relatively cheap, easy to manage, and generally accepted by patients. Studies have shown that women with short IPI do not know about birth spacing, know little about reliable contraceptives (49), and still face the risk of further intensive pregnancy. Therefore, for these women, more guidance is needed to help them properly arrange pregnancy time, promote the use of contraceptives and popularize reproductive health education. In China, a developing country, providing reasonable IPI recommendations through health education for postpartum women, and dietary guidance for pregnant women to improve nutritional status may be beneficial to preventing the occurrence of mental illnesses such as depression, and have a significant impact on public health.

## Conclusion

In this study, we found the five independent risk factors of third-trimester depression were gravidity, previous cesarean section delivery, folic acid, and vitamin D levels in early pregnancy and IPI. At the same time, we also proved the synergistic effects of short IPI and micronutrient deficiency on depression. Based on these findings, we recommend strengthening pre-pregnancy education to prolong and optimize pregnancy interval and emphasize the importance of perinatal nutritional assessment and appropriate intervention, and promote micronutrient supplements, when necessary, especially in pregnant women with short IPI.

## Strengths and limitations

The major advantage of this study is that it uses a female pregnancy-related cohort with detailed clinical information. The included subjects required that both deliveries were in our hospital, so the data variables, including maternal demographic information and delivery information, were reliably recorded in the medical archives. The sample size of the study is large, and the effects of parity and prenatal depression history were excluded. However, this study is a clinical retrospective cohort study, so it is difficult to avoid selection bias when selecting research objects. The research object of this study is limited to the research of specific ethnic groups of Chinese people, and the results should be carefully considered, because they cannot be extended to the whole population. We do not have information on postpartum factors such as postpartum examination, use of postpartum contraceptives, or breastfeeding, which may confuse the estimated impact of IPI and nutrients on adverse pregnancy outcomes (50, 51). In future studies, we can focus on examining the long-term effects of specific micronutrient consumption and pregnancy interval on neural development in animal models, which may provide important insights into the etiology and mechanism of perinatal depression.

## Data availability statement

The original contributions presented in this study are included in the article/supplementary material, further inquiries can be directed to the corresponding author.

## Ethics statement

The studies involving human participants were reviewed and approved by the Ethics Committee of the International Peace Maternity and Child Health Hospital (reference number GKLW 2021-23). The patients/participants provided their written informed consent to participate in this study.

## Author contributions

WG participated in research design, data collection, interpretation, and manuscript revision. JL participated in the design of the study, analyzed the data, and drafted the manuscript. YZ was responsible for collecting and interpreting data. WG and JL conceived the study and reviewed the manuscript. All authors contributed to the article and approved the submitted version.

## Footnotes

1
http://www.r-project.org
